# Diagnostic Role of Complete Blood Count (CBC) and C-reactive Protein (CRP) in Suspected Early-Onset Sepsis and Antibiotic Stewardship in the Neonatal Intensive Care Unit (NICU)

**DOI:** 10.7759/cureus.112999

**Published:** 2026-07-20

**Authors:** Hardik Shah, Nidhi Modi, Dhanani Vishakha Mansukhlal, Sushmita Devi Haodijam, Jaykumar Rameshbhai Jasani, Naiya Bhavsar, Varunkumar Gurjar, Hardik Mangroliya

**Affiliations:** 1 Department of Pediatrics, Dr. N.D. Desai Faculty of Medical Science and Research Centre, Dharmsinh Desai University, Nadiad, IND; 2 Department of Pediatrics, Shija Academy of Health Sciences, Imphal, IND; 3 Department of Pediatrics, Jasani Children Hospital, Surat, IND; 4 Department of Respiratory Medicine, Dr. N.D. Desai Faculty of Medical Science and Research Centre, Dharmsinh Desai University, Nadiad, IND; 5 Department of Pediatrics, Vibrant Multispeciality Hospital, Mehsana, IND; 6 Department of Emergency Medicine, Shree Satyay Hospital and ICU, Surat, IND

**Keywords:** antibiotic stewardship, complete blood count, c-reactive protein, early-onset sepsis, neonatal sepsis

## Abstract

Background: Early-onset sepsis (EOS) is a major cause of neonatal morbidity and mortality, but its diagnosis remains challenging because early clinical manifestations are nonspecific and blood culture results are not immediately available. Consequently, many neonates receive empirical antibiotics despite subsequently having negative blood cultures, increasing unnecessary antibiotic exposure and prolonging neonatal intensive care unit (NICU) stay. Readily available laboratory markers such as complete blood count (CBC) parameters and C-reactive protein (CRP) are commonly used to support clinical decision-making; however, neither marker alone is sufficiently accurate to confirm or exclude EOS.

Objective: This study evaluated the diagnostic utility of CBC parameters and CRP in neonates with suspected EOS and assessed their potential role in supporting antibiotic stewardship decisions.

Methods: This single-center retrospective observational study was conducted over one year in a NICU. Preterm and term neonates with EOS risk factors and National Neonatology Forum sepsis scores ≥10 were included, started on empirical antibiotics after blood culture collection, and monitored clinically. CBC parameters, CRP, and blood culture results were analyzed. Neonates with positive blood cultures were classified as culture-positive EOS, while those with negative cultures were classified as culture-negative suspected EOS.

Results: Among 573 NICU admissions, 94 neonates (16.4%) had suspected EOS, of whom 22 (23.4%) were culture-positive, and 72 (76.6%) were culture-negative. Leukopenia (WBC <5,000/mm^3^), a higher neutrophil percentage (>50%), thrombocytopenia, and elevated CRP were significantly associated with culture-positive EOS (p < 0.05). Gram-negative organisms predominated among culture-positive isolates (73%). Culture-negative suspected EOS accounted for most suspected cases and was associated with a longer NICU stay.

Conclusion: CBC parameters and CRP are useful adjuncts in evaluating suspected EOS when interpreted with blood culture results and clinical stability. These laboratory findings may inform antibiotic stewardship protocols, although their effect on antibiotic discontinuation requires prospective evaluation. Antibiotic decisions should be guided by clinical status, culture findings, serial laboratory trends, and local microbiological patterns.

## Introduction

Neonatal sepsis is still a significant contributor to neonatal deaths and morbidity in low- and middle-income countries. A systematic review of paediatric and neonatal sepsis revealed the burden of paediatric and neonatal sepsis worldwide and the importance of early diagnosis, management, and context-specific prevention strategies [1]. Early-onset sepsis (EOS) typically happens in the first 72 hours after birth and is not easily diagnosed because symptoms are vague and nonspecific and can mimic those of noninfectious neonatal illnesses. Early antibiotic therapy is crucial if sepsis is strongly suspected and is associated with poor in-hospital outcomes in neonatal intensive care units (NICUs) [2]. Neonatal diagnostic uncertainty is highest for preterm and/or clinically unstable neonates. Clinical manifestations can be confused with infection, such as respiratory distress, temperature instability, feeding intolerance, perinatal stress, metabolic disturbances, and prematurity-related complications. Antibiotic treatment is usual in cases of symptomatic preterm neonates, but data from trials indicate that treatment should not be continued for any nonspecific clinical signs [3]. According to major reviews, none of the clinical criteria or routine laboratory investigations are reliable at the time of presentation to exclude or confirm neonatal sepsis, and serial assessment, blood culture, and cautious interpretation are necessary [4].

Appropriate antibiotic prescribing in the newborn is a significant stewardship issue. There is a mismatch between suspected and proven bloodstream infection, as evidenced by NICU data, where antibiotic exposure is more common than proven bloodstream infection [5]. The clinical dilemma is in initiating antibiotic therapy early in the neonate who has a significant risk of sepsis but who does not have a positive culture, a clinical history of infection, or serial laboratory changes to support the diagnosis of sepsis. The implementation of risk-based screening and structured EOS recognition pathways can enhance the detection of antimicrobial use while maintaining safety [6]. The growing problem of antimicrobial resistance underscores the importance of evidence-based stewardship in neonatal care. The global action plan on antimicrobial resistance by the World Health Organization emphasizes public health priorities of optimized antimicrobial use, antimicrobial surveillance, and antimicrobial infection prevention and stewardship [7]. In neonates, the detrimental effects of unneeded antibiotics extend beyond resistance. Early-life antibiotics can impact the developing microbiome, and early changes to the microbiome have been associated with developmental, immune, and metabolic consequences [8]. Prevention of unnecessary antibiotic use within the first week of life is thus clinically relevant.

Management of neonates with suspected EOS and negative blood cultures remains challenging. Empirical antibiotics are often continued despite negative culture results because of concerns regarding false-negative cultures, persistently abnormal inflammatory markers, or ongoing clinical symptoms. Cantey and Baird criticized the unproven, routine use of antibiotics for culture-negative sepsis, suggesting that the use of antibiotics for longer periods without microbiological evidence might be associated with unnecessary exposure to antibiotics without proven benefit [9]. Klingenberg et al. defined culture-negative EOS as a "sweet spot" between efficient sepsis treatment and over-treatment, highlighting the importance of balancing early treatment with over-treatment [10]. The use of biomarker-guided strategies has been tested to reduce the duration of antibiotics in the treatment of suspected EOS. Randomized multicenter evidence has shown that the use of procalcitonin (PCT) for decision-making has decreased antibiotic exposure [11]. Use of routine PCT testing may be constrained in resource-limited NICUs, however, due to cost, availability, and turnaround time. Other markers, such as complete blood count (CBC) indices and C-reactive protein (CRP), are still in use, but their significance is more related to serial changes and stability of the patient than to absolute values.

However, recent evidence has cast doubt upon routine management based on routine CRP in EOS evaluations. The de-implementation of routine CRP testing resulted in less use of antibiotics among neonates at risk for EOS, indicating that an unnecessary CRP measurement may extend treatment [12]. In a large study, the limited usefulness of CRP as a diagnostic tool and inconsistent evidence of improved outcomes with its use were demonstrated, which may lead to a change in management [13]. Elevated CRP and white blood cell counts are also not reliable indicators of early-onset infection in term neonates [14]. Limiting CRP use has resulted in decreased unnecessary antibiotic exposures, thus providing an aid to the interpretation of results using selective biomarkers [15].

Combining CBC parameters with serial CRP measurements may partly overcome the limitations of either marker used alone. CBC abnormalities may appear early, whereas CRP rises later during the inflammatory response. A reassuring CBC pattern together with persistently low serial CRP values, negative blood cultures, and clinical stability may provide useful negative evidence against EOS and support discontinuation of empirical antibiotics at 36-48 hours. Reducing unnecessary antibiotic exposure is important because prolonged treatment may disrupt the neonatal microbiome, promote antimicrobial resistance, and increase treatment-related morbidity. An integrated biomarker-based approach may therefore strengthen antibiotic stewardship in the NICU, although CBC and CRP should not replace blood culture results or clinical judgment.

Objective of the study

This retrospective NICU-based study aimed to compare hemoglobin, total white blood cell count, neutrophil percentage, lymphocyte percentage, platelet count, and CRP between neonates with culture-positive EOS and those with culture-negative suspected EOS. Secondary objectives were to describe culture positivity, bacterial isolates, antibiotic susceptibility patterns, NICU stay, discharge status, and mortality. The relevance of these findings to antibiotic stewardship was explored descriptively rather than assessed as a formal outcome.

## Materials and methods

Study design and setting

This investigator-initiated, single-center, retrospective observational study was conducted in the NICU of Smt. S.M.S. Multispeciality Hospital, Dr. M.K. Shah Medical College and Research Centre, Ahmedabad, Gujarat, India, from January 2022 to December 2022. The study was approved by the institutional review board and ethics committee. As this was a retrospective review of routinely collected clinical and laboratory data, the ethics committee waived the requirement for informed consent.

Study population

All neonates admitted to the NICU during the study period who had maternal, perinatal, or clinical risk factors for EOS were screened using the previously published National Neonatology Forum (NNF) Sepsis Scoring System, as described in the NNF Clinical Practice Guideline on Diagnosis and Management of Neonatal Sepsis [16]. The scoring system was used as part of the institutional protocol to identify neonates requiring sepsis evaluation and initiation of empirical antibiotics. The NNF sepsis scores documented prospectively during routine clinical care were retrieved retrospectively from the medical records for the present analysis. As the NNF guideline is publicly accessible through the NNF clinical practice guideline platform, no separate permission was required for its use in this retrospective record-based study. EOS was considered in neonates presenting within the first 72 hours of life or those evaluated for sepsis based on perinatal risk factors. Neonates with an NNF sepsis score of ≥10 were included in the analysis as suspected EOS cases. Neonates with incomplete clinical records or missing blood culture reports were excluded from the study. For individual laboratory analyses, neonates with unavailable baseline CBC, CRP, or other laboratory parameters were excluded only from that specific analysis, and available-case denominators were reported where applicable. Neonates already receiving antibiotics before blood culture collection were also excluded.

NNF Sepsis Scoring System

The NNF Sepsis Scoring System has been previously described in NNF neonatal sepsis guidance and was cited accordingly in this article [16]. During routine clinical care, the NNF Sepsis Scoring System was applied prospectively by the treating team to support sepsis evaluation and decisions regarding empirical antibiotic initiation. For the present retrospective study, the documented NNF scores and corresponding clinical data were retrieved from the medical records. As no questionnaire, survey instrument, or copyrighted patient-reported tool was administered prospectively for research purposes, no separate permission was required for data collection. The scoring criteria are presented in Table 1.

**Table 1:** NNF Sepsis Scoring Criteria NNF: National Neonatology Forum; NICU: neonatal intensive care unit

No.	Criteria	Score
1	Intrapartum vaginal examination >3 times	6
2	Clinical chorioamnionitis	6
3	Prolonged rupture of membranes >12 hours	6
4	Maternal fever or sepsis within the preceding seven days	3
5	Home delivery	3
6	Preterm labor or premature onset of labor	3
7	Stay outside the NICU for >24 hours	3
8	Male sex	3
9	Birth weight <1.2 kg	2
10	Gestational age <30 weeks	2

Neonates with a total NNF sepsis score <10 were managed according to routine clinical assessment and institutional protocol and were not included in the present analysis. Neonates with a total score ≥10 underwent CBC, CRP, and blood culture testing before initiation of empirical first-line antibiotics. Accordingly, the study cohort comprised the 94 neonates with suspected EOS (NNF score ≥10) who fulfilled the study inclusion criteria.

Clinical management and data collection

For all included neonates, blood culture was collected before the initiation of empirical antibiotics in accordance with the institutional neonatal blood culture collection protocol. CBC and CRP were measured at approximately 24 hours of life or at the time of sepsis evaluation, depending on the timing of admission and clinical presentation. Repeat CBC, CRP, and blood culture were performed when there was clinical deterioration, persistent hemodynamic instability, or inadequate response to initial treatment. Antibiotic escalation to second-line therapy was based on clinical status, laboratory trends, and blood culture or antibiotic susceptibility results.

Data were extracted retrospectively using a structured proforma. The recorded variables included sex, gestational age, birth weight, mode of delivery, antenatal and perinatal risk factors, NNF sepsis score, CBC parameters, CRP level, blood culture result, isolated organism, antibiotic susceptibility pattern, duration of NICU stay, discharge status, and mortality.

Operational definitions

Suspected EOS was defined as the presence of maternal, perinatal, or clinical risk factors for sepsis with an NNF sepsis score ≥10. Culture-positive. EOS was defined as suspected EOS with growth of a pathogenic organism on blood culture. Culture-negative suspected EOS was defined as suspected EOS with no growth on blood culture. This term was used instead of “no sepsis” because a negative blood culture does not completely exclude infection owing to the imperfect sensitivity of blood culture and the possibility of evolving clinical illness. Prolonged NICU stay was defined as NICU admission lasting more than 14 days.

Laboratory parameters

The hematological and inflammatory parameters analyzed included hemoglobin, total white blood cell count, platelet count, neutrophil percentage, lymphocyte percentage, and CRP. Institutional laboratory reference ranges used for interpretation are shown in Table 2.

**Table 2:** Reference Ranges for Laboratory Parameters Values outside these ranges were categorized as abnormal according to institutional laboratory standards.

Parameter	Reference Range
Hemoglobin	11-14 g/dL
WBC count	10,000-26,000/mm^3^
Platelet count	150,000-450,000/mm^3^
Neutrophils	10%-50%
Lymphocytes	20%-40%
CRP	<6 mg/L

Outcome measures

The primary outcome was the difference in CBC parameters and CRP levels between neonates with culture-positive EOS and those with culture-negative suspected EOS. Secondary outcomes included the proportion of culture-positive EOS among suspected EOS cases, the proportion of culture-negative suspected EOS, distribution of bacterial isolates, antibiotic susceptibility patterns, duration of NICU stay, discharge outcome, and mortality.

Sample size

No formal a priori sample size calculation was performed because this retrospective study used a census-based approach and included all eligible neonates admitted during the predefined one-year study period. Of 573 NICU admissions, 94 neonates met the inclusion criteria and constituted the final analytical sample.

Statistical analysis

Data were analyzed using appropriate descriptive and inferential statistical methods. Continuous variables were summarized as the mean with standard deviation for normally distributed data and median with interquartile range for non-normally distributed data. Categorical variables were presented as frequencies and percentages. Comparisons between culture-positive EOS and culture-negative suspected EOS groups were performed using the independent-samples t-test or Mann-Whitney U test for continuous variables and the chi-square test or Fisher’s exact test for categorical variables, as appropriate. Test statistics corresponding to each comparison were calculated and included wherever applicable. A p-value of <0.05 was considered statistically significant.

## Results

Study cohort

During the one-year study period, 573 neonates were admitted to the NICU. Of these, 94 neonates (16.4%) met the criteria for suspected EOS according to the NNF Sepsis Scoring System and were included in the analysis. Among the suspected EOS cases, 22 neonates (23.4%) had culture-positive EOS, while 72 neonates (76.6%) had culture-negative suspected EOS (Table 3).

**Table 3:** Distribution of NICU Admissions and Suspected Early-Onset Sepsis Cases NICU: neonatal intensive care unit

Variable	Number (%)
Total NICU admissions	573
Suspected sepsis	94 (16.4)
Culture-positive sepsis	22 (23.4)
Culture-negative suspected sepsis	72 (76.6)

Demographic characteristics

Among the 22 neonates with culture-positive EOS, 14 (63.6%) were male, and eight (36.4%) were female. In the culture-negative suspected EOS group, 32 neonates (44.0%) were male and 40 (56.0%) were female. Normal vaginal delivery was more common in the culture-positive EOS group, occurring in 13 neonates (59.0%), whereas lower segment cesarean section was more common in the culture-negative suspected EOS group, occurring in 47 neonates (64.0%).

Birth weight differed significantly between the two groups. The mean birth weight was 1.868 ± 0.406 kg in the culture-positive EOS group and 1.491 ± 0.463 kg in the culture-negative suspected EOS group (t = 3.43, p = 0.001). Gestational age also differed significantly, with a mean gestational age of 36.4 ± 2.854 weeks and 34.17 ± 3.922 weeks, respectively (t = 2.47, p = 0.015) (Table 4).

**Table 4:** Demographic and Clinical Characteristics of Neonates With Suspected Early-Onset Sepsis Continuous variables were compared using the independent-samples t-test. Categorical variables were compared using the chi-square test. Test statistics are reported as t values for continuous variables and χ^2^ values for categorical variables. A p-value <0.05 was considered statistically significant. NICU: neonatal intensive care unit

Variable	Culture-positive sepsis, n = 22	Culture-negative suspected sepsis, n = 72	Statistical test	Test statistic	p-value
No. of newborns	22 (23.4%)	72 (76.6%)	-	-	-
Gender					
Male	14 (63.6%)	32 (44.0%)	Chi-square test	χ^2^ = 2.48	0.115
Female	8 (36.4%)	40 (56.0%)			
Mode of delivery					
Normal vaginal delivery	13 (59.0%)	25 (34.0%)	Chi-square test	χ^2^ = 4.16	0.042
Lower-segment cesarean section	9 (41.0%)	47 (64.0%)			
Birth weight category					
<1.5 kg	4 (18.0%)	49 (68.0%)	Chi-square test	χ^2^ = 17.46	<0.001
1.5-2.0 kg	12 (54.0%)	16 (22.0%)			
2.0-2.5 kg	5 (22.0%)	5 (7.0%)			
>2.5 kg	1 (4.5%)	2 (8.0%)			
Birth weight, mean ± SD	1.868 ± 0.406 kg	1.491 ± 0.463 kg	Independent-samples t-test	t = 3.43	0.001
Gestational age category					
<34 weeks	4 (18.0%)	35 (49.0%)	Chi-square test	χ^2^ = 20.64	<0.001
34-37 weeks	5 (22.0%)	28 (39.0%)			
>37 weeks	13 (59.0%)	9 (13.0%)			
Gestational age, mean ± SD	36.4 ± 2.854 weeks	34.17 ± 3.922 weeks	Independent-samples t-test	t = 2.47	0.015
Outcome					
Discharged	19 (86.0%)	64 (89.0%)	Chi-square test	χ^2^ = 0.10	0.747
Death	3 (14.0%)	8 (11.0%)			
NICU stay category					
>14 days	6 (28.0%)	70 (92.0%)	Chi-square test	χ^2^ = 53.26	<0.001
<14 days	16 (72.0%)	2 (8.0%)			
NICU stay, mean ± SD	17.2 ± 5.823 days	21.95 ± 5.635 days	Independent-samples t-test	t = -3.43	0.001

Clinical outcomes were comparable between the two groups (χ^2^ = 0.10, p = 0.747). Prolonged NICU stay of >14 days was significantly more frequent in the culture-negative suspected EOS group than in the culture-positive EOS group (χ^2^ = 53.26, p < 0.001). The mean NICU stay was 17.2 ± 5.823 days in the culture-positive EOS group and 21.95 ± 5.635 days in the culture-negative suspected EOS group (t = -3.43, p = 0.001).

The longer NICU stay observed in the culture-negative suspected EOS group should not be interpreted as evidence of greater infection severity. This group had significantly lower gestational age and birth weight and included a higher proportion of very low-birth-weight and preterm neonates. Their prolonged hospitalization was therefore likely influenced by prematurity-related monitoring, feeding support, respiratory care, thermoregulation, and other noninfectious neonatal morbidities. As these potential confounders were not adjusted for in a multivariable analysis, the association between culture status and duration of NICU stay should be interpreted cautiously.

Risk factors for EOS

Prolonged rupture of membranes (PROMs) >12 hours was observed in 14 neonates (63.0%) with culture-positive EOS and 68 neonates (94.0%) with culture-negative suspected EOS. Preterm or premature onset of labor was present in nine culture-positive cases (41.0%), and 63 culture-negative suspected EOS cases (88.0%). Maternal fever or sepsis within the preceding seven days was present in two culture-positive cases (9.0%), and five culture-negative suspected EOS cases (7.0%). No neonates in either group had a history of more than three intrapartum vaginal examinations or clinical chorioamnionitis (Table 5).

**Table 5:** Risk Factors for Early-Onset Sepsis (EOS) Categorical variables were compared using Fisher’s exact test because of small cell frequencies. A p-value <0.05 was considered statistically significant. Statistical testing was not applicable when both groups had zero observations. NICU: neonatal intensive care unit; PROM: prolonged rupture of membranes

Risk factor	Culture-positive EOS, n = 22	Culture-negative suspected EOS, n = 72	Statistical test	p-value
PROM >12 hours	14 (63.6%)	68 (94.4%)	Fisher’s exact test	<0.001
Intrapartum vaginal examinations >3	0	0	Not applicable	-
Clinical chorioamnionitis	0	0	Not applicable	-
Maternal fever/sepsis within the preceding seven days	2 (9.1%)	5 (6.9%)	Fisher’s exact test	0.664
Home delivery	2 (9.1%)	4 (5.6%)	Fisher’s exact test	0.622
Preterm labor or premature onset of labor	9 (40.9%)	63 (87.5%)	Fisher’s exact test	<0.001
Stay outside the NICU for >24 hours	1 (4.5%)	4 (5.6%)	Fisher’s exact test	1.000
Male sex	14 (63.6%)	32 (44.4%)	Fisher’s exact test	0.146
Birth weight <1.2 kg	1 (4.5%)	2 (2.8%)	Fisher’s exact test	0.555
Gestational age <30 weeks	3 (13.6%)	7 (9.7%)	Fisher’s exact test	0.694

Hematological findings within 48 hours

Baseline laboratory data were available for 19 of the 22 neonates with culture-positive EOS and all 72 neonates with culture-negative suspected EOS; the laboratory comparisons were based on these available-case denominators. Within the first 48 hours, WBC count was significantly lower in neonates with culture-positive EOS than in those with culture-negative suspected EOS. Leukopenia, defined as WBC count <5,000/mm^3^, was present in 16 culture-positive cases (85.0%) compared with 10 culture-negative suspected EOS cases (14.0%). The mean WBC count was 4,380/mm^3^ in the culture-positive EOS group and 21,000/mm^3^ in the culture-negative suspected EOS group (p < 0.001).

Neutrophil percentage was significantly higher in the culture-positive EOS group. Neutrophils >50% were observed in 11 culture-positive cases (58.0%) compared with seven culture-negative suspected EOS cases (10.0%). The mean neutrophil percentage was 62.2% in the culture-positive EOS group and 48.2% in the culture-negative suspected EOS group (t = 3.89, p < 0.001). Platelet count also differed significantly between groups. Platelet count <150,000/mm^3^ was observed in 17 culture-positive cases, and 20 culture-negative suspected EOS cases. The mean platelet count was 138,000/mm^3^ in the culture-positive EOS group and 214,000/mm^3^ in the culture-negative suspected EOS group (t = -5.00, p < 0.001).

Biochemical findings within 48 hours

CRP was significantly higher in the culture-positive EOS group. Elevated CRP was observed in 18 culture-positive cases (95.0%), while only one culture-positive case (5.0%) had CRP <6. Among culture-negative suspected EOS cases, 68 neonates (94.0%) had CRP <6, and four (6.0%) had CRP between 6 and 40. The mean CRP level was 36.4 in the culture-positive EOS group and 2.1 in the culture-negative suspected EOS group (p < 0.001). Hemoglobin and lymphocyte percentages did not show statistically significant differences between the two groups. The mean hemoglobin level was 17.318 g/dL in the culture-positive EOS group and 17.43 g/dL in the culture-negative suspected EOS group (p = 0.857). The mean lymphocyte percentage was 32.3% in the culture-positive EOS group and 28.4% in the culture-negative suspected EOS group (p = 0.109).

Follow-up laboratory findings in culture-positive EOS

Baseline laboratory data were available for 19 of the 22 neonates with culture-positive EOS and all 72 neonates with culture-negative suspected EOS; laboratory comparisons were therefore based on these available-case denominators. On follow-up testing among the 19 culture-positive EOS cases with available laboratory data, improvement was observed in several laboratory parameters. The mean WBC count increased from 4,380/mm^3^ within 48 hours to 7,850/mm^3^ on follow-up; the mean neutrophil percentage decreased from 62.2% to 38.6%; the mean platelet count increased from 138,000/mm^3^ to 168,000/mm^3^; and the mean CRP decreased from 36.4 mg/L to 3.8 mg/L. Follow-up values were presented descriptively and were not included in the between-group statistical comparison (Table 6).

**Table 6:** Hematological and Biochemical Parameters Among Neonates With Culture-Positive EOS and Culture-Negative Suspected EOS Continuous variables were compared between culture-positive EOS cases with available laboratory data within 48 hours (n = 19) and culture-negative suspected EOS cases within 48 hours (n = 72) using the independent-samples t-test. Test statistics are reported as t values. Follow-up values for culture-positive EOS cases are presented descriptively to demonstrate laboratory trends and were not included in the between-group statistical comparisons. A p-value <0.05 was considered statistically significant. EOS: early-onset sepsis; WBC: white blood cell; CRP: C-reactive protein

Parameter	Category/summary	Culture-positive EOS within 48 hours, n = 19	Culture-positive EOS at follow-up, n = 19	Culture-negative suspected EOS within 48 hours, n = 72	Statistical test	Test statistic	p-value
Hemoglobin	<11 g/dL	1 (5.0%)	2 (10.0%)	3 (4.0%)	Independent-samples t-test	t = -0.16	0.857
	12-14 g/dL	2 (10.0%)	3 (16.0%)	5 (7.0%)			
	15-20 g/dL	12 (63.0%)	13 (68.0%)	46 (64.0%)			
	>20 g/dL	4 (21.0%)	1 (5.0%)	18 (25.0%)			
	Mean	17.318	16.36 ± 2.673	17.43			
WBC count	<5,000/mm³	16 (85.0%)	1 (5.0%)	10 (14.0%)	Independent-samples t-test	t = -15.25	<0.001
	5,000-10,000/mm³	2 (10.0%)	10 (53.0%)	13 (18.0%)			
	10,000-24,000/mm³	1 (5.0%)	7 (39.0%)	48 (67.0%)			
	>24,000/mm³	0	1 (5.0%)	1 (1.3%)			
	Mean	4,380/mm³	7,850/mm³	21,000/mm³			
Neutrophils	<10%	0	0	1 (1.3%)	Independent-samples t-test	t = 3.89	<0.001
	10%-50%	8 (42.0%)	11 (58.0%)	64 (89.0%)			
	>50%	11 (58.0%)	8 (42.0%)	7 (10.0%)			
	Mean	62.2%	38.6%	48.2%			
Lymphocytes	<20%	1 (5.0%)	0	1 (1.3%)	Independent-samples t-test	t = 1.56	0.109
	20%-40%	16 (84.0%)	15 (79.0%)	66 (92.0%)			
	>40%	2 (10.0%)	3 (16.0%)	5 (7.0%)			
	Mean	32.3%	38.4%	28.4%			
Platelet count	<50,000/mm³	3 (16.0%)	0	1 (1.3%)	Independent-samples t-test	t = -5.00	<0.001
	50,000-150,000/mm³	14 (74.0%)	2 (10.0%)	19 (26.0%)			
	>150,000/mm³	2 (10.0%)	17 (89.0%)	52 (72.0%)			
	Mean	138,000/mm³	168,000/mm³	214,000/mm³			
CRP	<6 mg/L	1 (5.0%)	16 (84.0%)	68 (94.0%)	Independent-samples t-test	t = 7.00	<0.001
	6-40 mg/L	12 (63.0%)	3 (16.0%)	4 (6.0%)			
	40-60 mg/L	3 (16.0%)	0	0			
	>60 mg/L	3 (16.0%)	0	0			
	Mean	36.4 mg/L	3.8 mg/L	2.1 mg/L			

Blood culture isolates

Among the 22 culture-positive EOS cases, six isolates (27.0%) were Gram-positive organisms, and 16 isolates (73.0%) were Gram-negative organisms. Among Gram-positive isolates, four were methicillin-sensitive *Staphylococcus aureus*, one was methicillin-resistant *S. aureus* (MRSA), and one was *Enterococcus* species. Among Gram-negative isolates, eight were *Escherichia coli*, four were *Acinetobacter* species, and four were *Klebsiella* species. *E. coli* was the most common isolate overall.

Gram-positive antibiotic susceptibility pattern

Among Gram-positive isolates, methicillin-sensitive *S. aureus* showed 100% sensitivity to penicillin, fluoroquinolones, cotrimoxazole, meropenem, colistin, and chloramphenicol. Sensitivity to piperacillin-tazobactam, tetracycline, doxycycline, and linezolid was 75%. The MRSA isolate showed sensitivity to fluoroquinolones, piperacillin-tazobactam, cotrimoxazole, meropenem, colistin, tetracycline, doxycycline, linezolid, and chloramphenicol. The *Enterococcus* isolate showed sensitivity to cefoperazone-sulbactam, piperacillin-tazobactam, cotrimoxazole, meropenem, colistin, tetracycline, doxycycline, linezolid, and chloramphenicol (Table 7).

**Table 7:** Gram-Positive Blood Culture Isolates and Antibiotic Susceptibility Pattern MRSA: methicillin-resistant *Staphylococcus aureus*

Antibiotic	Methicillin-sensitive *Staphylococcus aureus*, n = 4	MRSA-1	*Enterococcus*-1
Penicillin	4(100%)	0	0
Fluoroquinolones	4(100%)	1(100%)	0
Cephalosporin	2(50%)	0	0
Gentamicin	1(25%)	0	0
Amikacin	1(25%)	0	0
Cefoperazone-Sulbactam	2(50%)	0	1(100%)
Piperacillin-tazobactam	3(75%)	1(100%)	1(100%)
Cotrimoxazole	4(100%)	1(100%)	1(100%)
Meropenem	4(100%)	1(100%)	1(100%)
Colistin	4(100%)	1(100%)	1(100%)
Tetracycline	3(75%)	1(100%)	1(100%)
Doxycycline	3(75%)	1(100%)	1(100%)
Linezolid	3(75%)	1(100%)	1(100%)
Chloramphenicol	4(100%)	1(100%)	1(100%)

Gram-negative antibiotic susceptibility pattern

Among Gram-negative isolates, *E. coli* was the most frequent isolate, followed by *Acinetobacter* species and *Klebsiella* species. *Acinetobacter* species showed 100% sensitivity to cotrimoxazole, meropenem, colistin, linezolid, and chloramphenicol. *E. coli* showed 100% sensitivity to cotrimoxazole and meropenem, and *Klebsiella* species showed 100% sensitivity to cotrimoxazole, meropenem, linezolid, and chloramphenicol. Reduced sensitivity to penicillin and cephalosporins was observed across gram-negative isolates (Table 8).

**Table 8:** Gram-Negative Blood Culture Isolates and Antibiotic Susceptibility Pattern

Antibiotic	*Acinetobacter* species, n = 4	*Escherichia coli*, n = 8	*Klebsiella* species, n = 4
Penicillin	1(25%)	3(37.5%)	1(25%)
Fluoroquinolones	2(50%)	5(62.5%)	2(50%)
Cephalosporin	1(25%)	3(37.5%)	1(25%)
Gentamicin	3(75%)	6(75%)	2(50%)
Amikacin	1(25%)	3(37.5%)	2(50%)
Cefoperazone-Sulbactam	2(50%)	7(87.5%)	2(50%)
Piperacillin-tazobactam	2(50%)	6(75%)	1(25%)
Cotrimoxazole	4(100%)	8(100%)	4(100%)
Meropenem	4(100%)	8(100%)	4(100%)
Colistin	4(100%)	7(87.5%)	3(75%)
Tetracycline	2(50%)	4(50%)	2(50%)
Doxycycline	2(50%)	4(50%)	2(50%)
Linezolid	4(100%)	7(87.5%)	4(100%)
Chloramphenicol	4(100%)	6(75%)	4(100%)

Overall, culture-positive EOS accounted for 23.4% of suspected EOS cases. Gram-negative organisms predominated, representing 73.0% of culture-positive isolates. WBC count, neutrophil percentage, platelet count, and CRP differed significantly between culture-positive EOS and culture-negative suspected EOS. Hemoglobin and lymphocyte percentages were not significantly different between the two groups. Culture-negative suspected EOS represented the majority of suspected EOS cases and was associated with longer NICU stay.

## Discussion

This study aimed to assess the diagnostic utility of CBC parameters and CRP in neonates with suspected EOS, with a focus on culture-proven infection and antibiotic stewardship. Culture-positive EOS accounted for a minority of suspected cases, highlighting the common discrepancy between clinically suspected and microbiologically confirmed neonatal sepsis. In the present cohort, leukopenia, thrombocytopenia, elevated CRP, and a higher neutrophil percentage were more frequently observed among culture-positive cases. The observed increase represented relative neutrophilia, based on the differential leukocyte percentage rather than the absolute neutrophil count. Although neutropenia is more commonly associated with severe EOS, neutrophil responses may vary with the timing of sample collection, gestational age, perinatal stress, and the stage of infection. Early inflammatory responses may be associated with transient increases in neutrophil percentage, whereas neutropenia may develop later due to increased peripheral consumption or bone marrow exhaustion. Since absolute neutrophil count and immature-to-total neutrophil ratio were not evaluated, this finding should be interpreted cautiously and not considered a standalone diagnostic indicator. These findings are consistent with those of Ognean et al., who reported that CBC and differential parameters are most useful when interpreted alongside clinical assessment rather than as standalone screening tests [17].

These biomarkers may have clinical implications beyond initial diagnosis. After empirical antibiotics are initiated for suspected EOS, decisions regarding continuation should incorporate blood culture results, clinical progression, and repeat laboratory findings. In the present study, follow-up CBC parameters and CRP showed descriptive improvement among culture-positive cases; however, no formal interval-based efficacy analysis was performed. In contrast, Liu et al. reported that WBC count, neutrophil percentage, and CRP had limited clinical value for monitoring antibiotic response across the assessed intervals, indicating that these markers should not be relied upon independently for treatment monitoring [18].

The predominance of culture-negative suspected EOS highlights the potential relevance of antibiotic stewardship in neonatal care. This group had a longer NICU stay, although this finding was likely influenced by lower gestational age, lower birth weight, and prematurity-related care needs rather than infection severity alone. Structured protocols combining clinical assessment, blood culture results, and serial laboratory findings have been associated with reduced antibiotic use and shorter hospitalization in term neonates with suspected EOS [19]. In the present study, CBC parameters and CRP were evaluated as adjunctive markers; their effect on antibiotic duration or discontinuation was not formally assessed.

It is important to remember the limitations of the biomarkers in these cases. A systematic review and meta-analysis of maternal, cord blood, and neonatal biomarkers revealed inconsistent performance in the diagnosis of EOS, with no single biomarker being sufficiently accurate for independent decision-making [20]. The same limitation is true of the present study. Leukopenia, thrombocytopenia, neutrophilia, and increased CRP were associated with culture-positive EOS, but must not be used in place of blood culture or clinical judgment. Their usefulness is in the context of risk stratification, especially in the absence of sophisticated biomarkers or the cost of advanced biomarkers.

Point-of-care and multimarker strategies may improve the diagnostic assessment of neonatal sepsis. Goyal et al. have investigated CRP, interleukin-6 (IL-6), and PCT in neonates with clinically suspected sepsis and found that multi-biomarker strategies might be superior to single biomarkers [21]. IL-6 and PCT were not included in the present study, which reduces the ability to compare with larger panels. In resource-limited NICUs, however, both CBC and CRP are still very relevant as they are inexpensive, readily available, and familiar to clinicians.

The diagnostic value of CBC-derived indices and CRP in neonatal sepsis workup has also been recently confirmed, with the caveat that these markers could be influenced by gestational age, birth weight, the degree of perinatal stress, and non-infectious inflammation [22]. This is relevant to the present study group, with a high proportion of low birth weight and pre-term babies. This could be part of the reason why some culture-negative infants have abnormal laboratory results and highlights the danger of overdiagnosing when biomarkers are used independently without a good clinical correlation.

Diagnosing culture-negative EOS remains challenging because clinical signs are nonspecific and currently available biomarkers have limited diagnostic accuracy [23]. Vasilescu et al. emphasized the need to interpret risk factors, clinical findings, and biochemical markers together rather than relying on a single test [23]. Published stewardship protocols suggest that discontinuation of empirical antibiotics may be considered at 36-48 hours in clinically stable neonates with negative cultures and no evidence of site-specific infection; this approach was not directly evaluated in the present study. Persistent clinical deterioration or worsening laboratory findings should prompt reassessment for occult infection or an alternative diagnosis.

PCT has been evaluated as a diagnostic marker for EOS in both high-resource and resource-limited settings [24]. Its routine implementation may still be restricted in some NICUs by cost, assay availability, and laboratory capacity. CBC parameters and CRP, therefore, remain accessible adjuncts, but they should not independently determine antibiotic duration. Clinical stability and negative cultures should remain the primary considerations, while serial sepsis-screen findings may provide supportive rather than decisive evidence. Persistently abnormal or worsening findings should prompt continued observation, repeat clinical assessment, and investigation for occult infection or an alternative diagnosis.

This study has some limitations. First, it was a retrospective single-center study, which may limit the generalizability of the findings to other NICU settings. Second, the number of culture-positive EOS cases was relatively small, limiting the statistical power of subgroup analyses. Third, advanced biomarkers such as PCT and IL-6 were not routinely available and therefore could not be evaluated alongside CBC parameters and CRP. Fourth, the retrospective dataset did not consistently capture important sepsis-related complications, including shock, fluid bolus or inotropic support, pneumonia, and disseminated intravascular coagulation, limiting assessment of illness severity beyond mortality and NICU stay. Finally, antibiotic duration and discontinuation were not directly measured, and no formal antibiotic stewardship intervention was evaluated. These limitations should be considered when interpreting the findings and planning future multicenter prospective studies.

## Conclusions

This study evaluated the clinical utility of CBC parameters and CRP in neonates with suspected EOS. Leukopenia, thrombocytopenia, a higher neutrophil percentage, and elevated C-reactive protein were more frequent in culture-positive cases. When interpreted alongside blood culture results and clinical status, these laboratory findings may inform the development of antibiotic stewardship protocols; their effect on antibiotic discontinuation requires prospective evaluation. These markers should remain adjunctive and should not replace clinical judgment or microbiological confirmation. Periodic review of local antibiograms and structured stewardship protocols may help reduce unnecessary antibiotic exposure and antimicrobial resistance in the NICU.

## References

[1] Fleischmann-Struzek C, Goldfarb DM, Schlattmann P, Schlapbach LJ, Reinhart K, Kissoon N (2018). The global burden of paediatric and neonatal sepsis: a systematic review. Lancet Respir Med.

[2] Schmatz M, Srinivasan L, Grundmeier RW (2020). Surviving sepsis in a referral neonatal intensive care unit: association between time to antibiotic administration and in-hospital outcomes. J Pediatr.

[3] Ruoss JL, Bazacliu C, Russell JT (2021). Routine early antibiotic use in symptomatic preterm neonates: a pilot randomized controlled trial. J Pediatr.

[4] Shane AL, Sánchez PJ, Stoll BJ (2017). Neonatal sepsis. Lancet.

[5] Schulman J, Benitz WE, Profit J (2019). Newborn antibiotic exposures and association with proven bloodstream infection. Pediatrics.

[6] van Herk W, Stocker M, van Rossum AM (2016). Recognising early onset neonatal sepsis: an essential step in appropriate antimicrobial use. J Infect.

[7] Flannery DD, Coggins SA, Medoro AK (2025). Antibiotic stewardship in the neonatal intensive care unit. J Intensive Care Med.

[8] Stiemsma LT, Michels KB (2018). The role of the microbiome in the developmental origins of health and disease. Pediatrics.

[9] Cantey JB, Baird SD (2017). Ending the culture of culture-negative sepsis in the neonatal ICU. Pediatrics.

[10] Klingenberg C, Kornelisse RF, Buonocore G, Maier RF, Stocker M (2018). Culture-negative early-onset neonatal sepsis: at the crossroad between efficient sepsis care and antimicrobial stewardship. Front Pediatr.

[11] Stocker M, van Herk W, El Helou S (2017). Procalcitonin-guided decision making for the duration of antibiotic therapy in neonates with suspected early-onset sepsis: a multicentre, randomized controlled trial. Lancet.

[12] Singh N, Gray JE (2021). Antibiotic stewardship in NICU: de-implementing routine CRP to reduce antibiotic usage in neonates at risk for early-onset sepsis. J Perinatol.

[13] Dhudasia MB, Benitz WE, Flannery DD (2023). Diagnostic performance and patient outcomes with C-reactive protein use in early-onset sepsis evaluations. J Pediatr.

[14] Liu L, Liu X, Zhang L, Ma J, Ding F (2025). High CRP and white blood cell counts are not reliable indicators of early-onset neonatal infection in full-term infants. Front Pediatr.

[15] Capone V, Venturelli S, Cresta E (2026). Restricting C-reactive protein use in early-onset neonatal sepsis reduces unnecessary antibiotic exposure. Antibiotics (Basel).

[16] (2026). National Neonatology Forum India. Diagnosis and management of neonatal sepsis - NNFI CPG Dec 2021. Dec.

[17] Ognean ML, Boicean A, Șular FL, Cucerea M (2017). Complete blood count and differential in diagnosis of early onset neonatal sepsis. Rev Romana Med Lab.

[18] Liu C, Zhang Y, Shang Y, Fang C, He Q, Xie L (2020). Clinical values of common biomarkers for efficacy monitoring of antibiotics in early-onset neonatal sepsis. Transl Pediatr.

[19] Gyllensvärd J, Ingemansson F, Hentz E, Studahl M, Elfvin A (2020). C-reactive protein- and clinical symptoms-guided strategy in term neonates with early-onset sepsis reduced antibiotic use and hospital stay: a quality improvement initiative. BMC Pediatr.

[20] van Leeuwen LM, Fourie E, van den Brink G, Bekker V, van Houten MA (2024). Diagnostic value of maternal, cord blood and neonatal biomarkers for early-onset sepsis: a systematic review and meta-analysis. Clin Microbiol Infect.

[21] Goyal M, Mascarenhas D, Rr P, Haribalakrishna A (2024). Diagnostic accuracy of point-of-care testing of C-reactive protein, interleukin-6, and procalcitonin in neonates with clinically suspected sepsis: a prospective observational study. Med Princ Pract.

[22] Narayanakar P, Metgud S, Bhandankar M (2019). Utility of hematological parameters and C-reactive protein levels in early diagnosis of neonatal sepsis. J Sci Soc.

[23] Vasilescu DI, Dan AM, Stefan LA, Vasilescu SL, Dima V, Cîrstoiu MM (2025). Assessment of culture-negative neonatal early-onset sepsis: risk factors and utility of currently used serum biomarkers. Children (Basel).

[24] El-Amin Abdalla EO, Salih FA, Salih HF, Elamin OE, Gamaleldin MA, Mustafa BM (2017). Procalcitonin in the diagnosis of early-onset neonatal infection in resource-limited settings. Cogent Med.

